# Association of QRS-T angle and Late Gadolinium Enhancement in patients with a Clinical Suspicion of Myocarditis

**DOI:** 10.7150/ijms.57010

**Published:** 2021-06-04

**Authors:** C.J. Jensen, B. Zadeh, J.M. Wambach, M. Lambers, K. Nassenstein, O. Bruder

**Affiliations:** 1 Department of Cardiology and Angiology, Contilia Heart and Vascular Center, Elisabeth-Krankenhaus Essen, Essen, Germany.; 2 Ruhr University Bochum, Bochum, Germany.; 3 Department of Diagnostic and Interventional Radiology and Neuroradiology, University Hospital Essen, Essen, Germany.

**Keywords:** QRS-T angle, cardiovascular magnetic resonance, late gadolinium enhancement, fibrosis, necrosis, myocarditis

## Abstract

**Objective:** To investigate the association of a wide QRS-T angle on the surface ECG and late gadolinium enhancement on contrast-enhanced cardiovascular magnetic (CMR) imaging in patients with clinically suspected myocarditis.

**Background:** Diagnosis and risk stratification in patients with suspected myocarditis is particularly challenging due to a great spectrum of clinical presentations. Late gadolinium enhancement (LGE) visualizes myocardial necrosis and fibrosis in patients with biopsy-proven myocarditis. The presence or absence of late gadolinium enhancements in these patients is prognostically meaningful. The QRS-T angle from the surface ECG, on the other hand, may serve as a simple and easily available risk marker in suspected myocarditis.

**Methods:** We enrolled 97 consecutive patients that were referred to CMR imaging for a clinical suspicion of myocarditis. All patients obtained a standardized digital 12-lead ECG for the calculation of the QRS-T angle and underwent contrast-enhanced CMR imaging. Patients were divided into two groups according to the absence or presence of LGE on CMR.

**Results:** 78 of 97 patients with suspected myocarditis had LGE on CMR. Patients with LGE had wider QRS-T angles as compared to the patient group without LGE (53.95-47.5 vs. 26.2-21.2; p<0.001). The sensivity, specificity, negative predictive value and positive predictive value for a QRS-T angle above 90 degrees for LGE positive myocarditis were 16.5%, 100%, 24.7%, and 100%, respectively.

**Conclusion:** A wide QRS-T angle of 90 degrees or more is linked to myocardial fibrosis or necrosis (late gadolinium enhancement) in patients with suspected myocarditis.

## Introduction

Diagnosis and risk stratification in patients presenting with acute myocarditis is especially challenging due to the great heterogeneity of clinical presentation. Disease progression ranges from mild chest discomfort with undisturbed left ventricular function to severe dyspnea and progressive heart failure or even sudden cardiac death 1-4.

The QRS-T-angle can be easily derived from the digital routine surface ECG. It represents the difference in the vectors of depolarization (QRS loop) and repolarization (T wave). Several observational studies in a variety of cardiac diseases have shown that the QRS-T-angle is a strong and independent prognosticator of adverse cardiac events including sudden cardiac death 5, 6.

Late gadolinium enhancement (LGE) on contrast-enhanced cardiac magnetic resonance (CMR) imaging depicts myocardial necrosis and later replacement fibrosis (scar) in patients with myocarditis with high spatial resolution. LGE in myocarditis has been validated against histology 7, indicates severe disease 8 and has a strong prognostic impact in patients with clinically suspected 9 or biopsy-proven myocarditis 10.

Aim of this study was to investigate a possible association between an abnormal QRS-T-angle on the routine surface ECG and myocardial necrosis as visualized by LGE in patients with a clinical suspicion of myocarditis.

## Methods

We prospectively enrolled 97 consecutive patients that underwent CMR at the Cardiovascular MRI Center, Department of Cardiology and Angiology, Elisabeth Hospital Essen, Germany for clinically suspected myocarditis. The indication for CMR was left the discretion of the referring physician, however was strong enough to qualify for CMR imaging. In general clinical presentations were anginal or pericarditic chest pain, symptoms of heart failure such as dyspnea or edema and more unspecific complaints such as fatigue or palpitations. Patients obtained a standardized 12-lead digital ECG on the day of admission at a referring hospital or using a Schiller Cardiovit AT 102 plus®. Computerized values of QRS and T-wave axis were given by the Schiller AT 102 plus® software. The frontal QRS-T-angle was calculated as the absolute difference between the frontal QRS- and frontal T-wave axes. With a value greater than 180°, 360° were subtracted in order to give a continuous variable ranging from 0° to 180°.

CMR Imaging was performed on a 1.5 Tesla MR System (Magneto Avanto, Siemens Medical Solutions, Erlangen, Germany). Sequences were acquired using a phased array receiver coil during end-inspiratory breathholding and in line with the recommendations of the Society of Cardiovascular Magnetic Resonance (SCMR) 11. Contiguous short axis and three long axis CMR cine images were obtained using a steady state free precession (SSFP) sequences. For Late Gadolinium Enhancement (LGE) imaging at least 10 minutes following the i.v. administration of 0.15 mmol/kg gadoterate meglumine corresponding segmented inversion recovery gradient echo sequences (IR-GRE) short axis views were prescribed every 10mm (slice thickness 6mm) covering the entire left ventricle constantly adjusting the inversion time as described previously 12. In-plane resolution was typically 1.2 × 1.8 mm.

Cine and LGE images were evaluated masked to clinical data by two experienced observers. LV function was analyzed by outlining epicardial and endocardial borders on the short axis SSFP sequences. Papillary muscles were excluded from analysis. Left ventricular volumes and ejection fraction were derived from contour summation. The extent of late gadolinum enhancement was assessed on long and short axis contrast images by using the 17-segment model of the LV. To minimize the risk of overseeing LGE areas in short-axis images, long-axis cross correlation was performed routinely.

### Statistics

Continuous data were presented as means ± standard deviation and categorical variables were presented as numbers and percentages. The distribution of QRS-T angle data was found to be non-normal distributed using the Kolomorov-Smirnov and Shapiro-Wilk test. Therefore, comparisons between groups of continuous variables were performed using the non-parametric Withney-U test. Categorical variables were compared using the chi-square test. All tests were two-tailed and a p-value less than 0.05 was considered statistically significant.

Correlation of LGE as percentage of LVmass and the QRS-T angle was analysed using the two-sided non-parametric spearman-rho test. Receiver operating characteristic (ROC) analyses was performed to assess the diagnostic ability of the QRS-T angle greater than 90° to detect myocardial fibrosis in contrast-enhanced CMR. The association of the QRS-T angle and myocardial fibrosis in contrast-enhanced CMR was analyzed using a binary logistic regression model for a continuous variable and using the chi-square test for a categorical variable (QRS-T angle greater than 90°).

All statistical analyses were performed using IBM SPSS Statistics version 26.0 (IBM Corp., Armonk, N.Y., USA).

## Results

**Figure 1:**
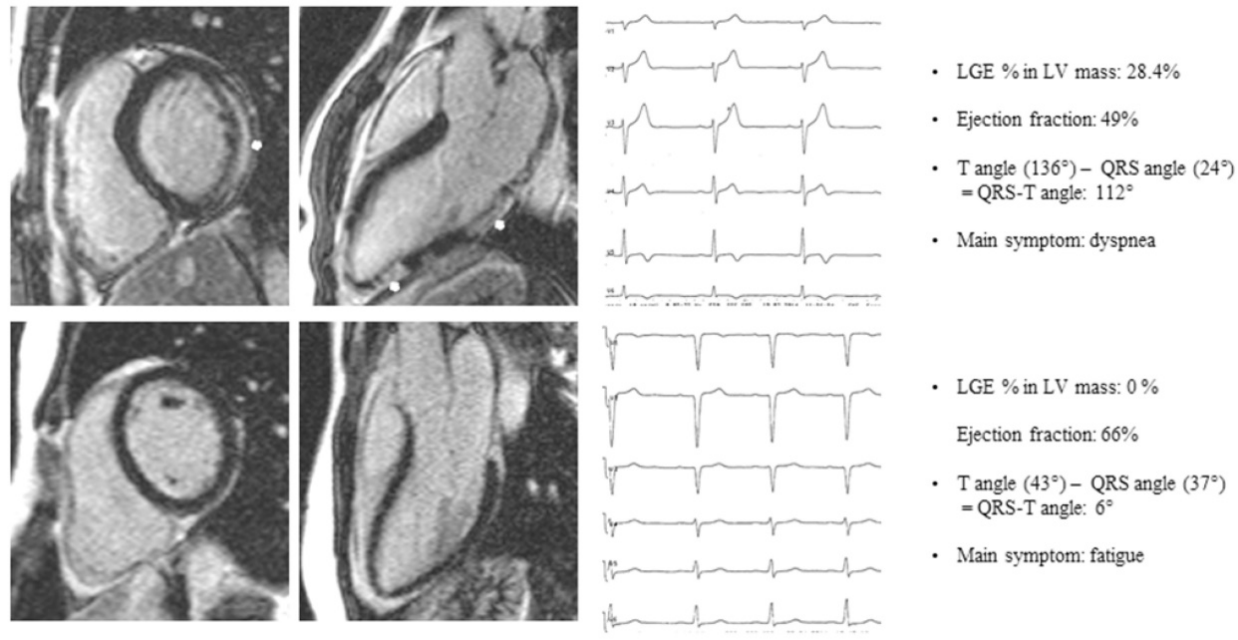
Patient A: basal end-diastolic short axis and 3-chamber long axis LGE image of a patient presenting with dyspnea, subepicardial LGE (white arrows), LGE % LV mass 28.4%, Ejection fraction 49%, QRS-T angle 112°. Patient B: basal end-diastolic short axis and 3-chamber long axis LGE image of a patient presenting with fatigue, no LGE, Ejection fraction 66%, QRS-T angle 6%. LGE late gadolinium enhancement.

In 97 patients (23 female, mean age 47.1-18.5 years) with an ejection fraction of 55.9-10.8% mean QRS-T angle was 48-45. 78 of 97 (80%) patients showed myocarditis type LGE on contrast-enhanced CMR, 19 of 97 (20%) patients had no signs of fibrosis on LGE CMR. Clinical examples are given in Figure 1. Analysing the data we found a moderate but highly significant correlation between LGE as percentage of LVmass and the QRS-T angle of 0.559, p<0.001. In patients with LGE QRS-T angle was greater than in patients without LGE (53.95-47.5 vs. 26.2-21.2; p<0.001). As a consequence of myocardial damage or more severe disease patients with LGE had a lower ejection fraction (54.6-11.6% vs. 60.7-4.3; p=0.016).

**Figure 2:**
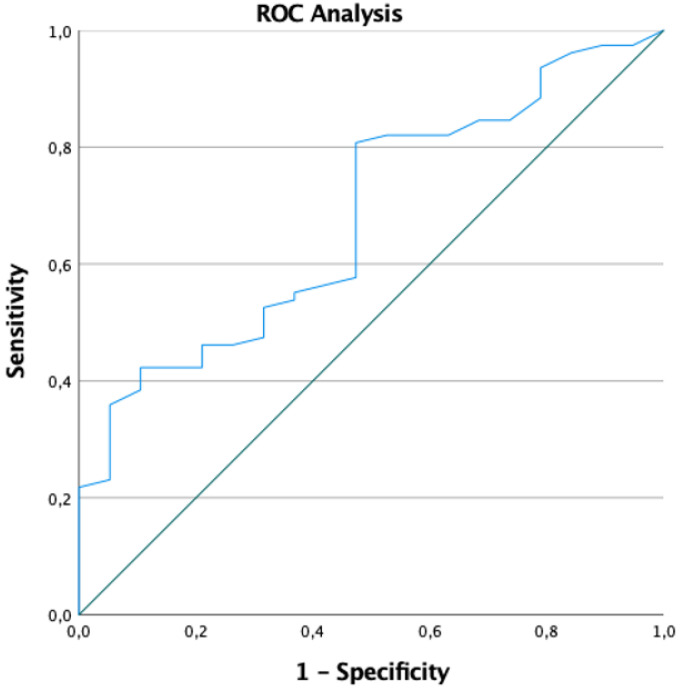
ROC analysis for a QRS-T angle greater than 90°.

All 16 patients with a QRS-T angle above 90 degree were LGE positive, there was no patient without LGE and a QRS-T angle above 90 degrees. The likelihood to detect LGE in contrast-enhanced CMR was 8.17 times higher in patients with a QRS-T angle greater than 90° (OR 7.18, p=0.037). In fact, the greater the QRS-T angle in surface ECG, the more likely was the presence of LGE in contrast-enhanced CMR (OR 1.022, CI 1.003;1.041, p=0.026). Area under the curve in ROC analyses for a QRS-T angle greater than 90° was 0.679 (95% Confidence interval: 0.554; 0.805, p=0.016) (Figure 2). The sensitivity, specificity, negative predictive value and positive predictive value for a QRS-T angle above 90 degrees for LGE positive myocarditis in this study population were 16.5%, 100%, 24.7%, and 100%, respectively.

## Discussion

Our study shows that in patients with suspected myocarditis an abnormal QRS-T angle of 90 percent or more relates to myocardial damage on contrast-enhanced CMR.

The QRS-T angle can be readily calculated from every routine surface ECG and may serve as a simple risk marker in various settings 5,6,13,14,15,16,17. LGE visualizes myocardial necrosis and fibrosis with high spatial resolution which in turn represents the substrate of conduction pathways for re-entrant ventricular tachycardia and predicts cardiac mortality and sudden cardiac death 18. So far only a few studies linked the QRST-T angle to the presence and extent of LGE in patients with ischemic cardiomyopathy 19, myocardial infarction 20, hypertrophic cardiomyopathy 21 and myocarditis 22.

Nonspecific ST-segment and T-wave abnormalities are common findings in patients with acute myocarditis and in a few cases even pericarditis-like ST-segment elevation occurs, but unfortunately the sensitivity of the electrocardiogram for myocarditis is low (47%) 23. In 587 patients with myocarditis Fischer et al. found an abnormal ECG in 78 percent 22; an abnormal ECG, the QRS-T angle and a QRS-T angle of 90 degrees or more were associated with a combined clinical endpoint on univariate analysis. ECG abnormalities were more frequent in patients with LGE. A combination of ECG parameters and LGE showed incremental prognostic power. Study inclusion was based on the diagnostic criteria suggested by the European Society of Cardiology Working Group on Myocardial and Pericardial Disease 24 including clinical presentations, ECG changes, troponine elevation and functional abnormalities on cardiac imaging.

In contrast inclusion in our study was driven by the clinical judgement of the referring physician, however clinical indication was obviously strong enough to justify advanced cardiac imaging. This approach reflects the real world of CMR as the absence of LGE rules out prognostically significant myocarditis 9,10. No advanced imaging techniques such as T1 mapping 25 were part of our CMR protocol, however advanced techniques are not yet standardized and are available only on a limited number of CMR scanners. In particular non contrast strain-encoded CMR could be of interest in this patient group as recent research indicates a promising role of strain imaging in the risk stratification of patients with heart failure 26. The concept behind contrast-enhanced CMR in myocarditis patients is that scar by LGE is the pathophysiological substrate of ventricular tachycardia and sudden cardiac death on the one hand and ventricular remodelling and progressive heart failure on the other hand. Against this background the proof of concept of any ECG parameter against LGE on CMR is particularly helpful. The QRS-T angle is such a promising ECG marker as the QRS-T angle can be easily calculated from every routine, digital surface ECG. The QRS-T angle represents the difference between the electrical vectors of depolarization and repolarization, which probably accounts for its prognostic potential in various clinical situations.

Given the diversity of clinical scenarios in patients with myocarditis and the fast, bedside and cheap availability of ECG, any ECG marker that relates to myocardial injury is extremely helpful. A wide QRS-T angle may serve as an additional marker that qualifies for CMR imaging to support initial diagnosis or justify more intensive follow-up. In our study cohort of 97 patients with clinically suspected myocarditis referred to CMR a wide QRS-T angle was linked to myocardial injury (LGE).

## Figures and Tables

**Table 1:** Baseline, ECG and CMR data stratified according to presence of myocardial fibrosis

Parameter	Study population, n= 97	Myocarditis with fibrosis, n= 78	Myocarditis without fibrosis, n= 19	p-value
Age (y)	47.1±18.5	46.2±17.6	50.8±21.1	0.123
Male gender (%)	74	78	59	0.029*
**Symptoms**				
Dyspnea (%)	34.1	46.4	21.8	0.012*
Angina (%)	20.4	14.1	22.5	0.394
Fatigue (%)	45.5	39.5	55.7	0.166
**EKG data**				
Heart rate (bpm)	82±17	76.7±20.9	84.3±16.5	0.435
QRS duration (ms)	98±17	93.2±17	88.9±6	0.234
QRS-T angle (°)	48±45	53.95±47.5	26.2±21.2	<0.001*
QRS-T angle ≥ 90°	16	16	0	
**MRI data**				
Ejection fraction (%)	59.9±10.8	54.6±11.6	60.7±4.3	0.016*
EDV (ml)	162.1±35.4	156.3±35.1	180.7±29.3	0.188
ESV (ml)	87.9±34.2	87.8±29.9	88.3±45.4	0.925
PE (mm)	3.4±3.2	3.7±3.1	2.25±2.9	0.09
Fibrosis % LV Mass	14.4±14.7	17.9±14.5	0.0±0.0	<0.001*

EDV end-diastolic volume, ESV end-systolic volume, PE pericardial effusion. * Highlights statistically significant differences between groups.
